# An empirical research on employee welfare and internal control quality

**DOI:** 10.1371/journal.pone.0290009

**Published:** 2023-08-11

**Authors:** Dongchuan Lin, Xuesong Tang, Hongyi Li, Guozhu He

**Affiliations:** 1 School of Accounting, Southwest University of Finance and Economics, Sichuan, China; 2 School of Business and Tourism, Sichuan Agricultural University, Sichuan, China; University of Almeria: Universidad de Almeria, SPAIN

## Abstract

Internal control is the key to achieve high-quality development of enterprises, but internal control failure cases frequently occur at home and abroad. Therefore, it is particularly important to explore ways to improve the quality of internal control and promote high-quality development of enterprises. Taking non-financial listed enterprises in China from 2015 to 2020 as research samples, this paper adopts empirical research methods to research the impact and mechanism of employee welfare on the quality of internal control of enterprises from the perspective of all-employee governance, aiming to explore ways for enterprises to improve the quality of internal control.The results showed that employee welfare can improve the quality of internal controls significantly. Furthermore, the effect is more significant in non-employee-intensive industries and high-marketization areas. Based on the analysis of internal control elements, the research found that employee welfare affects the quality of internal control mainly through two elements, risk assessment and control activities. However, the effects of employee welfare on the three other elements are insignificant. This paper enriched the relevant research on the economic consequences of employee welfare and the factors affecting the quality of internal control, and has certain enlightenment significance for the popularization of employee welfare, especially supplementary pension, and also provides a reference path for the improvement of the quality of internal control.

## 1. Introduction

A sound internal control system is the key to enhancing the risk management ability of enterprises and is the key breakthrough for the high-quality development of enterprises. However, the accounting scandal of Olympus in Japan, failure of internal control of MF Global in the United States, Zhangzi Island Incident and financial fraud in Kangmei Pharmaceutical in China and other internal control failures occurred frequently, which not only shows that there are still some problems in the effectiveness of internal control of some enterprises, but also arouses great attention to the internal control problems of enterprises around the world. It forced many countries to explore and innovate the reform of the internal control regulatory system, and directly led to the release of the SOX Act and Enterprise Risk Management—Integration Framework in the United States. Therefore, it is the common pursuit of all countries in the world to improve the quality of internal control and further promote the high-quality development of enterprises.

As the foundation of enterprise development and the main participants of internal control, employees play an important role in the effectiveness of internal control. The COSO Committee (1992) of the Financial Reporting Commission Against Fraud defined internal control as: "Internal control is a process, the process is affected by all personnel (including the board of directors, management and other personnel), its goal is to achieve the effectiveness and efficiency of the company’s operation, the reliability of financial reports, compliance to provide reasonable assurance", which emphasizes the universality of internal control. Rajan and Zingales also pointed out that the core issue of corporate governance is no longer limited to the analysis and discussion of the governance layer and management layer, but should include all employees including grassroots employees into the scope of governance (Rajan et al, 1998) [1]. The effectiveness of internal control is mainly investigated from two dimensions design and supervision. The management is mainly responsible for the design of internal control, and the operation of internal control mainly includes implementation and supervision. Among them, the implementation of internal control is mainly the responsibility of the employees, and the supervision of internal control is more responsible for the managers. Therefore, whether from the perspective of institutional norms or corporate practice, the effective operation of internal control requires the joint participation of all employees, including employees. However, previous research focused on the impact of governance and management on the quality of internal control, and few scholars take the grass-roots staff into consideration of the influencing factors of internal control quality.

Population aging is an important trend in the development of the world’s population and a major issue that countries need to face for a long time. According to data released by the Ministry of Internal Affairs and Communications in 2021, the number of elderly people aged 65 and over in Japan has reached a record high, and the elderly population aged 65 and above accounts for 29.1% of the total population. Data released by the German Federal Statistical Office in 2021 also shows that twice as many people are reemployed after retirement in Germany as 10 years ago. The continuous increase of the elderly population will not only increase the pension burden of the family and increase the financial pressure of the government, but also intensify the contradiction between supply and demand of the pension system, posing a huge challenge to the sustainability of the pension security system in various countries. Under the situation of actively responding to the aging of the population, it is urgent to improve the existing endowment insurance system and give full play to the supplementary role of enterprise annuity to the endowment insurance system. The United States and the United Kingdom passed corresponding laws in 2006 and 2008 respectively to implement the automatic accession mechanism of enterprise annuity, requiring qualified employees to join the enterprise annuity plan. China’s Enterprise Annuity Measures also came into effect in February 2018. In addition, enterprises also actively respond and continuously strengthen the benefits of employees. More and more enterprises incorporate additional welfare systems such as enterprise annuity, supplementary pension and supplementary medical care into incentive practice.

Based on this, this research will explore whether the additional supplementary pension and supplementary medical care system provided by enterprises have an impact on the quality of internal control from the perspective of all employees. Based on the two dimensions of the internal and external environment, this paper introduces two moderating variables: internal environmental factor (employee intensity) and external environmental factor (marketization process), and further analyzes whether the effect of employee welfare on the quality of internal control is heterogeneous under different employee intensity and marketization processes. In addition, according to the overall framework of internal control proposed by COSO (2014), the internal control composition includes five elements. This paper will further study the mechanism of employee welfare affecting the quality of internal control from the specific element level, and finally, put forward countermeasures and suggestions from the aspects of strengthening internal control construction and optimizing employee incentives.

Compared with the previous research, the main contributions of this paper are as follows: First of all, different from the previous research based on a single subject perspective, this paper starts from the perspective of full employees, explore the impact of comprehensive incentive policies of supplementary pension and supplementary medical care, which will affect both the current incentive perception and future real welfare benefits on the internal control, explore the new path to improve the quality of internal control of enterprises in our country which enriched the internal control quality factors and employee welfare economic consequences related research. Secondly, this study provides empirical data for the investigation of micro-governance effects of macro-policies such as supplementary pension and supplementary medical care, so as to help enterprises reasonably and effectively choose employee incentive measures to give full play to employee governance effects and further provide policy basis for the government to improve the endowment insurance policy and do a good job in top-level design.

## 2. Prior literature

### 2.1 Influencing factors of internal control

The previous research on the influencing factors of internal control quality can be summarized as internal environmental factors and external environmental factors. According to the existing research, scholars focus more on the internal environment of the enterprise, because the internal environment has a more direct impact on the quality of internal control. First, the characteristics of the enterprise, such as complexity, scale, financial situation, organizational change, internal control resources, and other basic characteristics (Doyle J T, 2007; rice S C, 2012) [2,3] have an impact on internal control to a certain extent. Specifically, the more complex the enterprise’s business activities are, the worse the financial situation is, in the period of organizational change, the rapid growth rate, and the lack of available resources for internal control, all of which lead to lower quality of internal control. With the further expansion of the research, scholars began to research from the perspective of corporate governance. The first is the governance layer. The characteristics of the audit committee, for example, the number of financial experts on the Audit Committee, frequency of Audit Committee meetings (Krishnan, 2007) [4], the internal audit capability, the assurance level of internal audit quality control, the follow-up process and the audit committee’s participation in reviewing the internal audit plan and results can all have a certain impact on the quality of internal control (Oussii et al, 2018) [5]. the chief audit executive (CAE) plays an important role in internal control as the principal person in charge of internal audit, and position turnover of CAE is often accompanied by a decline in the quality of internal control, and when the succeeding CAE has lower financial expertise than the former, the decline is even more pronounced (Gerald J. et al. 2022) [6]. The existence of institutional investors can have a certain supervisory effect on enterprises, and when an enterprise has qualified foreign institutional investors (Zhe Li. et al. 2021) [7]. In addition, the size of the board of directors (Jensen M C, 1993) [8] and the proportion of independent directors (Fama E F et al, 1983) [9] also have an impact on internal control. The second is managers characteristics. Executives play an important role in the construction of internal control, and executives with academic experience are more capable of designing and effectively implementing governance mechanisms to positively influence the effectiveness of internal control and improve the quality of internal control (Zhang K, 2020) [10]. In addition, external incentives such as board ownership (Kobelsky K, 2013) [11] can also have an impact on the quality of internal controls. From the perspective of the external environment, such as business environment, media supervision, financial analysts, external auditors, supervision and market factors also have a significant impact on the quality of internal control of enterprises to a certain extent. For instance, the process of marketization, the legal system, and media attention are significantly positively correlated with the effectiveness of internal control (Zhao Yuanxian, 2014) [12]. In addition, the longer the tracking period of financial analysts (Mao M Q, 2015) [13] and the longer the tenure of external auditors (Khlif H, Samaha K, 2016) [14], the more conducive to improving the quality of internal control.

### 2.2 Research on employee governance

With the penetration of the “people-oriented” concept, academia and enterprises gradually realize the importance of employees. Sustainable human resource management (SHRM) views employees as a very important resource for the organisation, while paying close attention to their preferences, needs, and perspectives (Sypniewska, 2023) [15]. Based on the fact that employee turnover is a global problem, scholars have found that cross training of employees can help reduce employee turnover (S. Arunkumar et al, 2012) [16]. In addition to reducing their attrition rate, effective management measures or incentives are key to enabling employees to play an effective governance role. As far as existing research is concerned, scholars have mostly focused on individual employees themselves, especially focusing on the motivation of employee well-being, employee satisfaction, employee performance, and employee engagement. Through a survey of 318 Ecuadorian workers, Loor found that CSR practices can have a positive impact on employees’ intrinsic motivation and trust in the organization, and further confirmed the mediating role of these two variables in the relationship between CSR and organizational commitment (Loor-Zambrano, 2022) [17]. Bastida also found that job discretion also affects employee subjective well-being (SWB), and this effect is greater for men. Moreover, job discretion is not homogeneous for SWBS at different levels of education, and the higher the level of education, the higher the level of SWB (Bastida, 2022) [18]. Moslehpour explores six ways to improve employee satisfaction. Each pathway includes perceived mission statement quality, romantic management philosophy, psychological combination of moral climate, low tendency towards moral ambiguity, and managers’ emotional competence. Among them, managers’ emotional competence is a core condition for high job satisfaction (Moslehpour, 2022) [19]. Based on data from five hospitals in Ghana, Hannah found that abuse of oversight by leaders can reduce employee engagement and thus detriment to the effective implementation of employees’ work (Hannah et al,2022) [20]. While incentives for employees can increase employee engagement and thus their performance (Siswanto. S et al, 2021) [21]. In addition, as an important manager of an enterprise, the words and actions of CEOs can also have a certain impact on employees, and research has found that a reputable CEO can have a positive impact on employees, which in turn increases employee engagement (Linjuan Rita Men, 2012) [22]. The advent of the information age has made the requirements of modern enterprises more stringent on the quality of employees, and the information literacy of employees not only conforms to the inevitable requirements of the development of modern enterprises, but also enhances the creativity of employees to a certain extent (Chang, CP et al, 2015) [23].

What are the economic consequences of employee involvement in corporate governance? From the perspective of incentives, if an enterprise enhances the status of employees and allows them to participate in management, equity incentives can not only supervise the private control interests of major shareholders and the privileged corruption of management, but also promote the improvement of enterprise innovation performance (Marianna M et al, 2006) [24]. Chang further showed that longer employee stock ownership periods and lower shareholding ratios were more likely to promote the level of innovation performance, and that employee stock options primarily incentivized innovation through risk-taking (Chang X et al, 2015) [25]. Previous research also found that high-wage employees tend to be more productive than low-wage workers, and this research shows that compensation incentives also contribute to the improvement of employee productivity (Roosaar, L et al, 2019) [26]. Moreover, specific to the economic consequences of employee welfare, research results have gradually begun to emerge in recent years. Lin Yuen (2021) [27] sorted and summed up the five insurance, retirement, allowance, education, subsidies and annuity, and other related expenditures to obtain the final employee welfare. Through empirical research, it was found that employee welfare can significantly improve enterprise performance. Zhang Dongxu (2021) [28] used the annuity plan payment data of A-share listed enterprises to empirically test the relationship between enterprise annuity in social security benefits and enterprise innovation performance. The results found that the implementation of an annuity plan can significantly improve enterprise innovation performance.

In summary, the academic community has made abundant achievements in the influencing factors of internal control. Scholars study the influencing factors of the internal and external environment (corporate characteristics, corporate governance) and the external environment (business environment, external audit tenure, etc.). Among the internal influencing factors of internal control, scholars have noticed that the characteristics and behaviors of senior executives have an impact on the quality of internal control, but few scholars have considered the role of junior employees in internal control and the combined effect of junior employees and senior executives. Moreover, as far as the current research results are concerned, scholars pay more attention to the motivation research to improve the performance of employee governance, while the research results of employee governance consequences are relatively few, on the basis of the existing research, this paper explores the impact on internal control of supplementary pension and supplementary medical care, which will not only affect the current incentive perception but also affect the real welfare treatment in the future.

## 3. Development of hypothesis

### 3.1 Employee welfare and internal control quality

Unlike equity incentives, which are mostly only for executives and core employees, employee welfare policy, as supplementary welfare, involves all employees of the enterprise and has an incentive effect on all ordinary employees. As direct participants in various business activities of an enterprise, employees play a vital role in improving enterprise performance and risk controll. Zhang Min (2014) [29] believes that effective employee governance is an important part of internal control and one of the important foundations for the effective operation of internal control. Employees can be self-motivated and self-disciplined, and the motivation for fraudulent behaviors can be weakened (Hochberg et al,2010) [30] by improving the welfare guarantee system of all employees, which can enhance the employees’ enthusiasm of internal control construction to a certain extent. The management will pay more attention to the long-term development of the enterprise, design an effective internal control system, and strictly supervise its implementation. At the same time, grass-roots employees are encouraged to actively participate in the implementation of the internal control and can play a supervisory role in the implementation of the internal control to a certain extent. The probability of corporate violations is effectively reduced, and the effectiveness of internal control is improved (Wang Ye et al, 2021) [31] through the cooperation of grassroots employees and managers. At the same time, Employees can be self-motivated and self-disciplined, and the motivation for fraudulent behaviors can be weakened (Luo Yanmei, 2020) [32] by improving the welfare guarantee system of all employees. In addition, good employee welfare can also greatly improve the opportunity cost of employee turnover, effectively reduce the employee turnover rate, save the turnover cost and also create a stable internal control environment. Based on this, this research expects that the implementation of employee welfare can significantly improve the quality of internal control. And the research hypothesis H1 is proposed.

H1: Employee welfare can improve the quality of internal control significantly.

### 3.2 Employee intensity, employee welfare, and internal control quality

A good employee incentive policy can contribute to improving the enterprise governance environment, alleviate the principal-agent problem between managers and ordinary employees to a certain extent, and provide a basic guarantee for continuous and effective internal control (Balsam S et al, 2014) [33]. At the same time, due to the differences in the intensity of employees in enterprises and the consideration of the balance between input and performance, the welfare input intensity of employees varies in different enterprises, resulting in different incentive effects.

Compared with high-employee-intensive enterprises, low-employee-intensive enterprises are mostly concentrated in mid-to-high-end industries, their profitability is relatively high (Shen Ruicheng et al., 2018) [34], and their employee base is relatively smaller. Therefore, enterprises with lower employee intensity are more capable of increasing the intensity of investment to enhance the convergence of the interests of employees and owners under the same conditions, which will be more able to increase the enthusiasm of employees to participate in corporate governance, so as to supervise and motivate all employees to participate in the formulation and effective implementation of internal control in an all-round and whole-process manner. Based on this, the research hypothesis H2 is proposed.

H2: The effect of employee welfare on the quality improvement of internal control will be more significant in enterprises with lower intensity of employees.

### 3.3 Marketization process, employee welfare, and internal control quality

As an organic unit that participates in and supports market economic activities, it is difficult to escape the influence and restrictions of a market-oriented environment for any enterprise. In recent years, the Chinese market economy has developed rapidly, and great achievements have been made in market-oriented reform. However, there are still regional differences and the level of marketization varies in different provinces, which will affect the governance behavior of micro-enterprises profoundly.

First of all, the government intervention is less in enterprises that are located in the higher-marketization regions where the degree of freedom of enterprises is higher, and the mobility of the labor force is stronger (Xia Lijun, 2007) [35]. Coupled with the fierce market competition environment, enterprises have stronger risk perception ability, and enterprises will show good governance behavior for obtaining a good competitive advantage in the market. Therefore, enterprises will pay more attention to the improvement of employee welfare policy and salary system, thus providing a good employee foundation for the improvement of internal control quality. Secondly, due to the high information transparency and relatively perfect legal system construction in the higher-marketization regions employees show a stronger awareness of rights protection, and the opportunistic behavior of enterprises is easier to be detected (Kim S H, 2013) [36], and the enterprises will face higher litigation cost. Therefore, enterprises will constantly improve employee welfare policies in order to make full use of the advantages brought by human capital, thus promoting the quality of internal control. Therefore, in areas with a high degree of marketization, whether it is based on passive market pressure, or actively improving employee welfare to obtain a human capital advantage, the result will lead to enterprise governance structure being optimized, and employees will be motivated to provide support and cooperation for the implementation of the internal control system, so as to promote the internal control quality effectively. Based on it, this paper proposes hypothesis H3.

H3: The effect of employee welfare on the quality improvement of internal control will be more significant in enterprises with a higher level of marketization process.

## 4. Research design

### 4.1 Sample selection and data sources

Considering the timeliness, continuity, and availability of data, basic samples are contained from listed companies in China’s main board market for the period 2015–2020, and further processes are as follows: (1) Eliminate ST, ST *, PT, and financial industries; (2) Eliminate abnormal data samples; finally, 4182 observation samples are obtained. The data of employee welfare in this paper comes from the enterprise ESG-CEGSG series data under the company’s characteristic database in the CNRDS database. The Internal Control Disclosure index comes from internal control and risk management series data in the DIB internal control database. The marketization process data comes from the “Chinese sub-province marketization index report (2018)” compiled by Wang Xiaolu and Fan Gang. And other data are derived from the National Bureau of Statistics and the Chinese Research Data Services Platform (CSMAR).

### 4.2 Variable definition

1. Explained variable: Internal control quality (ICQ)

This paper selects the internal control quality (ICQ) as the explained variable and selects the natural logarithm of the DIB internal control index as its proxy variable. The index system includes 5 first-level indicators and 65 second-level indicators. The higher the index means the higher the quality of internal control.

2. Explanatory variable: Employee welfare (EW)

The employee welfare (EW) data is directly derived from the data published by the CNRDS database. If the enterprise has a supplementary pension and supplementary medical system, the value is 1, otherwise, it is 0. This indicator reflects whether the enterprise has a good welfare policy.

3. Moderating variables: Employee intensity (EI), Marketization process (MP)
(1) Employee intensity (EI)

The data on employee intensity (EI) is derived from the CSMAR database. The number of employees at the end of the year is divided by the operating income of the year as the proxy variable for employee intensity. The higher the value means the higher the employee intensity. Based on it, the samples are grouped according to employee intensity. The employee intensity above the mean value is assigned 1, and the value below the mean value is assigned 0.

(2) Marketization process (MP)

This paper draws on the overall marketization index in “China ’s Provincial Marketization Index Report (2018)” compiled by Wang Xiaolu and Fan Gang to measure the marketization process. Since the index is only updated to 2016, it draws on the research of Yang Xingquan (2014) [37] to supplement the data from 2017 to 2020. The method of filling is as follows: The 2017 marketization index is the average of the 2016 index plus the average of the three-year index increases in 2014,2015 and 2016 relative to the previous year, and so on. On this basis, further processing is as follows: when the marketization index of the region is greater than the median, take 1 and when it is less than the median, take 0.

4. Control variables

Referring to the existing literature, this paper selects the listing years (FAGE), ownership concentration (TOP1), duality (DUAL), enterprise size (SIZE), the number of executives (EXE), the property rights (STATE), asset-liability ratio (LEV) and return on total assets (ROTA) as control variables. In addition, this paper also adds an industry dummy variable (IND) and annual dummy variable (YEAR). Specific variables descriptions are shown in Table 1.

**Table 1:** Variables table.

Variable type	Variable name		Variable meaning	Calculation method
Explained variable	ICQ		Internal control quality	taking the natural logarithm of the disclosure index of the DIB internal control information
Explanatory variable	EW		Employee welfare	Whether the company has the supplementary pension and supplementary medical care system, if yes, it is 1, or it is 0
Moderating variables	EI		Employee intensity	The number of employees at the end of the year divided by the operating income for the year
	MP		Marketization process	The general marketization index in the “Marketization Index Report by provinces in China (2018)”
Control variables	Enterprise Characteristics	SIZE	Enterprise size	The natural logarithm of total assets at year-end
		FAGE	Listing age	The number of years of the company from listing to the statistical date
	Enterprise governance	TOP1	Ownership concentration	The number of shares held by the largest shareholder divided by the total number of shares
		DUAL	Two-position integration	The chairman and general manager are the same people take 1, otherwise, take 0
		EXE	Number of executives	The number of senior executives in the company
		STATE	Nature of property right	The value of state-owned enterprises is 1, otherwise, it is 0
	Operating results	LEV	Assets-liability ratio	The total liabilities divided by total assets
		ROTA	Rate of return on total Assets	(Total profit+Financial expenses)/Total assets
Dummy variables	YEAR		Particular year	Annual dummy variable
	IND		Industry	Industry dummy variable

### 4.3 Model design

To study the effect of employee welfare on the quality of internal control, the following model is established:

$$ICQ_{it}=\beta_{0it}+\beta_{1}EW_{it}+\beta_{2}FAGE_{it}+\beta_{3}TOP1_{it}+\beta_{4}DUAL_{it}+\beta_{5}SIZE_{it}+\beta_{6}EXE_{it}+\beta_{7}STATE_{it}+\beta_{8}LEV_{it}+\beta_{9}ROTA_{it}+YEAR_{it}+IND_{it}+\varepsilon$$ (1)

In this model, i denotes sample enterprise and t denotes year; YEAR and IND represent year fixed effect and industry fixed effect respectively; ε represents the random disturbance term.

Furthermore, this paper conducts group regression on Model (1) according to EI and MP. To test whether the effect of employee welfare on the quality of internal control is heterogeneous due to different employee intensity and marketization processes.

## 5. Empirical results

### 5.1 Descriptive statistics

Table 2 shows the descriptive statistical results of the main variables. Among the 4182 samples, the mean of internal control quality (ICQ) is 5.215, the minimum is 3.417, and the maximum is 5.733, which indicates that the overall quality of internal control of the samples is high. The average value of employee welfare(EW) is 0.549, and the median is 5.256, which shows that the feature of the right-skewed distribution is obvious and 55.1% of listed enterprises in the sample have implemented supplementary pension and supplementary medical systems among the control variables, the average age of listing (FAGE) is 15.34, the maximum value is 30.05, and the minimum value is -2.05, indicating that the sample enterprises have a longlisting period as a whole, but there are still large differences among the enterprises. The average ownership concentration (TOP1) is 0.376, indicating that the shareholding ratio of the largest shareholder of the sample enterprises is below the medium level, and there is still a large gap among the enterprises. The average value of DUAL is 0.195, indicating that only 19.5% of the sample enterprises have the chairman and general manager concurrently. The number of executives (EXE) in the sample enterprises varies greatly, indicating that there are great differences in the governance structure of different enterprises. The average value of the asset-liability ratio (LEV) is 0.495, indicating that the solvency of sample enterprises is relatively good, and the overall level is appropriate. The average return on total assets (ROTA) is 0.057, indicating that the sample is in poor operating condition. The mean value of the intensity of employees (EI) is 0.152, and the median is 0, indicating that the overall employee intensity of the sample enterprises is low. The average value of the marketization process (MP) is 0.824, which indicates that most of the listed enterprises in the sample are in regions with a high marketization process and the overall level is on the high side. In general, the results of the descriptive analysis of control variables are consistent with those of Liu Ya (2021) [38] and Luo Yanmei (2020) [32].

**Table 2:** Descriptive statistics results.

Variable	N	mean	median	sd	min	max
ICQ	4182	5.215	5.256	0.222	3.417	5.733
EW	4182	0.549	1	0.498	0	1
FAGE	4182	15.34	16.63	7.18	-2.05	30.05
TOP1	4182	0.376	0.368	0.156	0.034	0.99
DUAL	4182	0.195	0	0.396	0	1
SIZE	4182	10.19	10.16	0.612	8.030	12.27
EXE	4182	7.024	7	2.660	0	22
STATE	4182	0.614	1	0.487	0	1
LEV	4182	0.495	0.505	0.189	0.0340	1.352
ROTA	4182	0.057	0.051	0.064	-0.725	0.484
EI	4182	0.153	0	0.360	0	1
MP	4182	0.824	1	0.381	0	1

### 5.2 Correlation analysis

As shown in Table 3, the correlation coefficient between employee welfare (EW) and internal control quality (ICQ) is 0.096, which is significantly positive at the level of 1%, that is, improving employee welfare can improve the quality of internal control of enterprises, which preliminarily verifies H1. The number of years the company has been listed (FAGE), enterprise size (SIZE), number of senior executives (EXE), return on total assets (ROTA), and marketization process (MP) are correlated positively with internal control quality (ICQ) at the level of 1% significantly, and the nature of property right (STATE) is correlated positively with internal control quality (ICQ) at the level of 10% significantly, indicating that it has a significant positive effect on the improvement of internal control quality. The two-position integration (DUAL) and employee intensity (EI) are correlated negatively with internal control quality (ICQ) at the level of 1% significantly, indicating that two-position integration and employee intensity have an inhibitory effect on internal control quality. Preliminary correlation test results are ideal, and further regression analyses were followed-up.

**Table 3:** Correlation analysis.

Variables	(1)	(2)	(3)	(4)	(5)	(6)	(7)	(8)	(9)	(10)	(11)	(12)
(1)ICQ	1											
(2)EW	0.096***	1										
(3)FAGE	0.079***	0.156***	1									
(4)TOP1	0.009	0.005	0.122***	1								
(5)DUAL	0.071***	0.071***	0.187***	0.064***	1							
(6)SIZE	0.173***	0.169***	0.163***	0.189***	-0.100***	1						
(7)EXE	0.067***	0.080***	0.011	0.007	0.007	0.226***	1					
(8)STATE	0.028*	0.141***	0.287***	0.323***	0.340**	0.213***	0.092***	1				
(9)LEV	0.020	0.075***	0.128***	0.058***	0.062***	0.543***	0.133***	0.153***	1			
(10)ROTA	0.044***	0.016	0.088***	0.056***	0.082***	0.010	0.001	0.162***	0.258***	1		
(11)EI	0.103***	0.098***	0.182***	0.003	0.081***	0.207***	0.004	0.082***	0.170***	0.033**	1	
(12)MP	0.084***	0.081***	0.036**	0.024	0.055***	0.058***	0.051***	0.035**	0.007	0.088***	0.002	1

*** p<0.01

** p<0.05

* p<0.1.

In addition, the variance inflation factor VIF value of the main variables is calculated, and the results are shown in Table 4. The VIF value of each variable is less than 1.7, which is far less than 10, indicating that there is no serious collinearity problem among variables.

**Table 4:** Test results for variance inflation factor (VIF) values.

Variable	VIF	1/VIF
EW	1.07	0.936
FAGE	1.23	0.814
TOP1	1.24	0.806
DUAL	1.15	0.869
SIZE	1.65	0.605
EXE	1.07	0.931
STATE	1.46	0.684
LEV	1.58	0.635
ROTA	1.15	0.869
EI	1.09	0.920
MP	1.02	0.977
MEAN VIF	1.25	

### 5.3 Main regression analysis

1. Employee welfare (EW) and internal control quality (ICQ)

In order to test the effect of employee welfare on the quality of internal control, model (1) is used for regression analysis. The results are shown in column (1) in Table 5. The regression coefficient of employee welfare (EW) is 0.015, which has a significant positive impact on the quality of internal control (ICQ) at the level of 5%, that is, the better the employee welfare, the higher the quality of internal control, H1 is verified. Through the implementation of the employee welfare policy covering all employees, the enterprises can motivate employees, so that employees are consistent with the goals of the enterprises and actively participate in the design and operation of internal control. In addition, employees play a certain supervisory role in the fraud and risk investment behavior of enterprises in order to protect their own interests due to their risk-averse tendency, so that enterprises can effectively avoid some risks and improve the internal control quality.

**Table 5:** Employee welfare and internal control quality.

Variable	Whole sample	High employee intensity	Low employee intensity	High marketization process	Low marketization process
	(1)	(2)	(3)	(4)	(5)
EW	0.015**	-0.012	0.018**	0.022***	-0.020
FAGE	-0.001	0.002	-0.001**	-0.001*	0.001
TOP1	-0.034	-0.186**	-0.006	-0.059**	0.039
DUAL	0.037***	-0.042	-0.034***	-0.032***	-0.053
SIZE	0.071***	0.031	0.067***	0.065***	0.079***
EXE	0.003***	0.006*	0.003**	0.002	0.012**
STATE	0.001	-0.004	-0.004	0.002	-0.004
LEV	0.109***	-0.131**	-0.092***	-0.100***	-0.170**
ROTA	0.001	-0.042	0.011	-0.040	0.055
IND	control	control	control	control	control
YEAR	control	control	control	control	control
N	4182	639	3543	3445	737
R^2^	0.208	0.260	0.224	0.206	0.296
Ajusted R^2^	0.193	0.187	0.207	0.189	0.232

2. Heterogeneity analysis of employee intensity (EI)

Columns (2) and (3) in Table 5 report the regression results of the samples of high and low-employee intensity enterprises. It can be seen that the regression coefficient of employee welfare in the high employee intensity group is not significant, while the regression coefficient of employee welfare in the low employee intensity group is 0.018, which has a significant positive impact on the quality of internal control at 5%, indicating that the implementation of good employee welfare policies in the low employee intensity enterprises can promote the internal control quality significantly, and H2 has been verified. Since China’s social insurance payment ratio is at a higher level than that of other countries, Chinese enterprises are also facing a heavier financial burden. If enterprises further implement additional supplementary pension and medical care systems for all employees, it means that enterprises will incur additional expenses beyond their legal obligations, which will aggravate the financial burden of enterprises. Moreover, compared with the low-employee-intensity enterprises, the high- enterprises may have a weaker implementation intensity of employee welfare enterprises due to their large employee base and low-profit level. Therefore, their incentive effect may be weaker than that of the enterprises with a small employee base. In this case, the formulation and effective implementation of internal control will also be adversely affected to a certain extent.

3. Heterogeneity analysis of marketization process (MP)

The regression results of the marketization process are shown in columns (4) and column (5) in Table 5. In the high marketization process group, the regression coefficient of employee welfare (EW) is 0.022, which has a significant positive impact on the quality of internal control (ICQ) at the level of 1%, while the impact of employee welfare on the quality of internal control is not significant in the low marketization process group, indicating that the implementation of good employee welfare policies plays a significant role in promoting the quality of internal control in areas with high marketization process, so H3 can be verified. The possible reason for this is that in areas with a high level of marketization process, the intense market competition environment encourages enterprises to formulate and implement employee welfare policies. At the same time, sound laws and regulations and high awareness of employee rights protection increase the litigation risk of enterprises, forcing enterprises to continuously improve the employee welfare system and pay more attention to the improvement of employee welfare policies and salary system. Under the influence of this marketization level, the active promotion and passive optimization of employee welfare behavior will greatly improve the level of employee governance and have a positive impact on the improvement of internal control quality.

4. Complementary analyses

Internal control includes five elements: internal environment, risk assessment, control activities, information and communication, and internal supervision. This paper further researches the mechanism of employee welfare (EW) on internal control quality (ICQ) from the perspective of internal control elements and deeply analyzes its internal relationship. As shown in Table 6, employee welfare is significantly positively correlated with risk assessment at the 5% level and is correlated positively with control activities at the 10% level significantly, while the internal environment, information and communication, and internal supervision are not significantly correlated with employee welfare. Therefore, employee welfare mainly affects the internal control quality through risk assessment and control activities.

**Table 6:** Employee welfare and internal control elements.

Variable	Internal environment	Risk assessment	Control activities	Information and communication	Internal supervision
EW	0.042	0.090**	0.144*	0.029	-0.046
FAGE	-0.003	-0.007**	0.023***	-0.015***	0.035***
TOP1	0.102	-0.205	0.435*	-0.548***	0.183
DUAL	-0.085*	-0.171***	-0.055	0.101**	0.030
SIZE	0.061	0.419***	0.250***	0.260***	0.553***
EXE	0.023***	0.009	0.034**	-0.005	0.002
STATE	0.003	-0.147***	-0.148	-0.216***	0.134
LEV	-0.233**	-0.191	0.820***	0.643***	-0.984***
ROTA	0.170	-0.694**	-0.216	-0.445*	0.588
IND	control	control	control	control	control
YEAR	control	control	control	control	control
N	4182	4182	4182	4182	4182
R^2^	0.079	0.218	0.077	0.338	0.107
Adjusted R^2^	0.062	0.203	0.059	0.325	0.090

As the basis of risk management, risk assessment is the key to the effectiveness of internal control. By improving employee welfare and implementing long-term supplementary pension and medical system welfare measures, enterprises make employees pay more attention to the long-term development of enterprises, recognize the importance of risk assessment more easily, and are willing to work for the long-term strategic goals of enterprises to provide support and cooperation for the implementation of enterprise risk assessment (Luo Yanmei, 2020) [32]. The employees will actively help enterprises to make effective risk assessment programs, promote the smooth development of risk assessment work, and strengthen the risk management capabilities of enterprises from the source, so as to improve the quality of internal control. The control activities refer to the procedures and means to formulate countermeasures according to the results of risk assessment to ensure the realization of internal control objectives. As the main participants of various business activities, employees have a natural connection with the design and implementation of control activities. Appropriate human resource policies and practices enable enterprises to have a certain number of capable and responsible employees, and high-quality employees can more effectively implement policies and procedures, make a rapid response after the risk occurs, help enterprise to establish and implement effective risk control mechanisms and strive to control risks within an acceptable range. In addition, As an important core strategic resource of enterprises, human resources play a vital role in enterprises (Chun et al, 2017) [39]. The implementation of the employee welfare policies can help optimize the human capital structure of the enterprises, encourage employees to participate in their work more actively and creatively, and makes them willing to work hard to achieve the long-term strategic goals of the enterprise. The control activities that penetrate the functions of all levels within the enterprise can be implemented more effectively, and ultimately the effectiveness of the overall internal control of the enterprise will be improved (Wang Ye et al., 2021) [31]. However, the establishment of internal environment construction, internal supervision mechanism, and information and communication channels may be more subject to the decision-making behavior of the senior leadership team of the enterprise, and less affected by the ordinary employees who occupy the vast majority of the enterprise. Therefore, the implementation of universal employee welfare has no obvious effect on it.

5. Robustness test
(1) PSM test

Considering the problem that employee welfare and internal control quality may be mutually causal, this paper adopts the propensity score matching method (PSM) to test the endogeneity in order to test the endogeneity of the sample. According to model 1, this paper takes the variable, the years of listing (FAGE), ownership concentration (TOP1), two-position integration (DUAL), enterprise size (SIZE), number of executives (EXE), nature of property rights (STATE), asset-liability ratio (LEV) and return on total assets (ROTA) as matching factors, and choose employee welfare (EWE) as the benchmark to process all samples by the method of PSM one-on-one matching in this paper. As shown in Table 7, the average effect t value of the matching post-processing group is 2.33, indicating that there is no significant difference between the samples before and after matching, and further passes the balance test, so the results of this paper are robust.

**Table 7:** PSM test results.

Variable	Sample	Treated	Controls	Difference	S.E.	T-stat
ICQ	Unmatched	5.234	5.191	0.043	0.007	6.22
	ATT	5.218	5.200	0.018	0.008	2.33
	ATU	5.192	5.226	0.034		
	ATE			0.027

(2) Variable replacement

Considering that there are other methods to measure the quality of internal control, this paper further uses the internal control index of Xiamen University as a measure of the quality of internal control. The regression results are shown in Table 8, indicating that employee welfare will significantly promote the quality of internal control, which is consistent with the above results. Therefore, the results of this study are robust.

**Table 8:** Regression results of replacing the explained variable.

Variable	(1)	(2)
EW	0.012***	0.009***
CONTROLS		control
IND		control
YEAR		control
Sample size	4182	4182
R^2^	0.006	0.193
Adjusted R^2^	0.006	0.177

(3) Model Replacement

To further verify the robustness of the regression results, this paper tests by changing the model. On the basis of processing the internal control quality (ICQ) data as a binary variable, the logit model is used for regression analysis. The results are shown in Table 9. After changing the model, the employee welfare (EW) and internal control (ICQ) in the whole sample are significantly positively correlated at the 5% level. The group regression of employee intensity (EI) and marketization process (MP) are also basically consistent with the above results, indicating that this empirical study has certain robustness.

**Table 9:** Replacement model regression results.

Variable	(1)	(2)
EWE	0.314***	0.206***
CONTROLS		control
IND		control
YEAR		control
Sample size	4182	4175

## 6. Discussion

This paper explores the impact of employee welfare on the quality of internal controls and found that employee welfare can have a positive impact on the quality of internal controls. There are similar articles, but the number is relatively small and the angles of the research are slightly different. On the one hand, scholars have paid attention to the important governance role of employees in enterprises. We find that existing studies have found the governance role of employees in enterprises, from employee stock ownership (Blasi, 2018; Bryson, 2019; Adwan, 2022) [40–42], salary incentives (Baliga, 1990; Sigler,2011) [43,44] and others have carried out research, and found that these factors can effectively mobilize the enthusiasm of employees, improve employee satisfaction, and thus improve enterprise performance. However, employee stock ownership plan and compensation incentive are only researched from a single perspective of long-term and short-term incentives.

On the other hand, with the advent of the aging society, the government’s financial burden has intensified, the sustainability of old-age security has been threatened, and a series of social problems have emerged, which has attracted the common attention of theoretical and practical circles, and many scholars have begun to study old-age insurance. Among them, the most concerned is the implementation of enterprise annuity. Research on the American capital market shows that, as an Inside Debt, enterprise annuity is conducive to restrain the excessive risk taking of senior executives, thus improving the investment efficiency of enterprises (Edmans,2011) [45]. Similar to Edmans’ research, we research the impact of integrated incentive policies (supplementary pension, supplementary medical care) on firms.As an important aspect of employee welfare, supplementary pension (enterprise annuity) is different from the previous single perspective based on short-term incentive or long-term incentive. We conduct research from the comprehensive incentive policy that affects not only the current incentive cognition but also the actual welfare benefit in the future. This is the difference between our research perspective and previous research.Different from Edmans’ research, we pay more attention to whether the implementation of employee welfare can effectively reduce the risks of enterprises and improve the quality of internal control of enterprises, and our research conclusions also support this argument. In addition, this research is also similar to the conclusions of Luo Yanmei (2020) [32] and Zhang Dongxu (2020) [28] on the effects of employee compensation incentive and employee equity incentive on internal control quality. The former found that short-term compensation incentive can promote the improvement of internal control quality. The latter finds that employee stock ownership can promote the improvement of internal control quality from the perspective of long-term incentives. It can be seen that both short-term and long-term employee incentives can have a certain impact on the quality of internal control. This paper explores the effects of comprehensive incentive policies (supplementary medical care and supplementary pension) on the quality of internal control from the perspective of employee welfare, which affects both current incentive cognition and future actual welfare benefits.

## 7. Conclusion and recommendation

### 7.1 Main conclusions and recommendation

In the context of frequent internal control failure incidents of listed companies in various countries, there are still many problems in internal control. This research studied the effect and mechanism of employee welfare on the quality of internal control based on the background of the implementation of supplementary pension and medical insurance systems. The results showed that employee welfare can improve the quality of internal controls significantly. Furthermore, the effect is more significant in non-employee-intensive industries and high-marketization areas. Based on the analysis of internal control elements, the research found that employee welfare affects the quality of internal control mainly through two elements, risk assessment and control activities. However, the effects of employee welfare on the three other elements are insignificant.

Based on the above research conclusions, this paper makes suggestions from two aspects: internal control construction and employee welfare optimization:

First, scientifically design the internal control system and supervise its effective operation. First of all, enterprises should scientifically and reasonably design their internal control system according to their internal and external environment and their own actual conditions. Secondly, improve the supervision mechanism of the internal control operation of the enterprise, include senior managers and grass-roots employees in the supervision mechanism, and build a top-down and bottom-up two-way supervision system to ensure the effective operation of internal control.

Second, implement differentiated employee welfare policies and give full play to the effect of employee governance. Enterprises should pay attention to supplementary welfare, especially the implementation of supplementary pension and supplementary medical policies, which is conducive to the effective implementation of the internal control system of enterprises. On the one hand, on the whole, enterprises should improve employee welfare policies, establish long-term, fair and reasonable incentive mechanisms, attach importance to the importance of supplementary benefits, and improve employee participation enthusiasm. At the same time, strengthen employees’ risk management awareness and ability, enhance risk identification and risk response levels, and improve the overall effect of employee welfare policies in promoting the improvement of internal control quality from the two dimensions of willingness and ability. On the other hand, enterprises should objectively judge their own situation, scientifically evaluate the effect of the total amount of employee benefits and unit strength, implement differentiated employee welfare policies according to the differences in employee density and marketization process, enhance the matching degree of employee benefits, and improve the marginal effect of employee welfare policies in promoting the improvement of internal control quality.

### 7.2 Theoretical contributions

The main theoretical significance of this paper is to enrich the research on the influencing factors of internal control and the economic consequences of employee governance, especially employee welfare, which can provide a certain reference for subsequent scholars’ research. This paper also has certain practical significance: from the perspective of all employees, this paper explores the impact of employee welfare (supplementary pension, supplementary medical treatment), which not only affects the current incentive perception but also affects the real welfare treatment in the future, on the quality of internal control of enterprises.

### 7.3 Practical implications

This research also has certain practical significance. First of all, the research conclusions obtained through data verification provide empirical evidence for investigating the micro-governance effects of macro policies such as supplementary pension and supplementary medical care. At the same time, this research also provides new ideas for improving the quality of internal control of enterprises, and provides a reference basis for the government to do a good job in policy formulation.

### 7.4 Limitations and future research

However, there are some limitations in this research. First of all, this research takes Chinese listed enterprises as the research object, and the selection of samples has certain limitations, due to the differences in national conditions of various countries, so the conclusions of this research may lack universality. Secondly, because the supplementary pension and supplementary medical care of Chinese enterprises have not yet been popularized in most enterprises, the availability of data is affected, and the specific implementation amount cannot be obtained, so this paper uses dummy variables to measure the quality of employee welfare with the implementation of supplementary medical care and supplementary pension, although most of the current research uses this measurement method, but it is undeniable that it will have a certain impact on the research.

With the continuous development of social economy, employees as the core resources of enterprises, their role in contemporary enterprises is becoming more and more prominent, and exploring their role in governance in enterprises will also be a continuous topic. On the basis of the existing research, in view of the shortcomings of the current research, more in-depth research can be carried out in the future: (1) collect employee welfare data in various countries, conduct comparative analysis of their implementation effects, and find out the commonalities and differences in various countries; (2) With the further improvement of information disclosure, gradually construct quantitative indicators of employee welfare and improve the shortcomings of existing research; (3) Explore which groups (executives, grassroots employees or all employees) can maximize their effectiveness by implementing employee benefits, so as to help enterprises do a good job in accurate governance. This can be used as the author’s follow-up research direction, and can also provide a certain reference for the follow-up research of other scholars.
